# Accounting for access to healthcare: Analyzing interview talk of hard-to-reach regions’ residents and mobile medical units’ professionals in Greece

**DOI:** 10.1177/13591053241266384

**Published:** 2024-07-27

**Authors:** Evangelia Sofia Vergouli, Lia Figgou, Panagiotis Koulouvaris, Nikolaos Scarmeas

**Affiliations:** 1 The Open University, UK; 2 Aristotle University of Thessaloniki, Greece; 3 National and Kapodistrian University of Athens, Greece; 4 Columbia University, New York, USA

**Keywords:** access to healthcare, critical discursive social psychology, health inequalities, primary healthcare initiatives, stigma against illness

## Abstract

This study explores how social actors account for psychosocial barriers to healthcare access. Interviews with 17 residents in remote regions of Greece and 12 professionals employed by Mobile Medical Units were analyzed using the tools and concepts of critical discursive social psychology. Analysis indicated that, oriented to different accountability concerns, residents tended to attribute reluctance to seek medical help to structural barriers, while professionals leaned toward psychological and individual-centered explanations. Findings also highlighted the construction of living in hard-to-reach areas as both a “cure” and a “curse” for residents’ capacity to achieve a healthy status, representing remote communities as both enhancing solidarity and social support and as promoting stigmatization against illness and social isolation. Building upon prior discourse-oriented approaches in health psychology, the study seeks to exemplify how a discursive and rhetorically oriented research agenda can be employed to explore how health inequalities are enacted and (re)produced in social interactions.

## Introduction

The present study explores how social actors account for psychosocial barriers affecting access to healthcare in interview discourse. The tools and methods of critical discursive social psychology (Wetherell, 1998) were employed to analyze interviews with citizens in Greece’s remote regions and professionals employed by the Mobile Medical Units (MMUs), an initiative addressing differential access to healthcare in Greece’s geographical landscape.

### Exploring disparities in health and social psychology: The contribution of qualitative studies

The study of health inequalities has been a significant area of interest in health and social psychology. However, by concentrating on identifying individual cognitions that might predict (un)healthy behaviors, psychologists often tend to conclude that people from lower socioeconomic backgrounds hold beliefs that result in them failing to achieve a healthy status (Day, 2012). Concomitantly, disparities in health between social groups are often viewed as the product of individual “health beliefs” that lead to poor life choices, the implication being that these beliefs are wrong and need to be altered (Hodgetts and Chamberlain, 2000).

The turn to language and discourse (e.g., Billig, 1982; Potter and Wetherell, 1987) and the emergence of critical social psychological approaches (Gough et al., 2013; IbáÍñez and Íñiguez, 1998; Parker, 2002) deeply affected psychological research into health and illness. Scholars in the field of critical health psychology (see Murray, 2014) expressed their dissatisfaction with the theories and methods that proliferated within mainstream health psychology, arguing that positivism’s emphasis on individualism and scientific neutrality not only discouraged meaningful political engagement and action that could potentially transform the conditions that foster poor health (Murray and Poland, 2006) but also undermined efforts to reduce health inequalities on local and national levels (Murray, 2012).

Substantive evidence confirms that inequitable social arrangements adversely affect individuals both materially and psychologically, through lived experiences of stigma, stress, loneliness, powerlessness, and poor-quality social relations (Hall and Lamont, 2013; Hodgetts et al., 2014; Kearns et al., 2015). Over the past 2 decades, several qualitative psychosocial studies have explored the impact of structural determinants on people’s health and health-related behaviors in various contexts. For instance, a UK-based study by Hodgetts et al. (2007) explored the connection between homelessness, social marginalization, and people’s health-related practices (see also Stolte and Hodgetts, 2015), revealing that stress and stigma were directly linked to material disadvantage and social exclusion. These findings align with Popay et al.’s (2003) observation that feeling out of place leads to distrust, stress, and stigma, which in turn are associated with increased risk of illness. In Granado et al.’s (2014) focus group study on the low uptake of mammography screening among Barbadian women, participants expressed concerns about the lack of public awareness and knowledge regarding the procedure, the fear of enduring the societal taboo of breast cancer and dealing with the potential cost of treatment following a diagnosis. Similarly, in Sanuade et al.’s (2021) focus group study, fear and concerns over financial constraints, healthcare workers’ corrupt practices, long queues, health system delays, and shortages in breast cancer specialists were reported as impediments to breast cancer treatment initiation among a Ghanian sample.

Several researchers who take a relational and community-orientated approach to health psychology (e.g., Campbell and Cornish, 2014; Campbell and Jovchelovitch, 2000; Campbell and Murray, 2004; Hodgetts et al., 2016) have advocated for the need to engage with situational and intergroup relations to cast light on the foundational causes of health inequalities and disparities in health behaviours and outcomes. Psychosocial research, such as Campbell’s (2003) study of a community-based intervention designed to raise awareness of the transmission of HIV in South Africa and De-Graft Aikins et al.’s (2020) social psychological participatory project aiming to build cardiovascular disease competence in a Ghanian community, highlights the need to strategically involve community members in decisions about health service design and delivery in order to address differential access to healthcare fairly. For such mobilization to occur, group members must come together to construct identities that become the basis for collective action (Campbell and Jovchelovitch, 2000; see also Cicognani et al., 2020).

A consolidated framework for making sense of how social groups and social identity-mediated mechanisms determine health constitutes the research agenda that has come to be known as ‘the social cure’ (after Haslam et al., 2009; Jetten et al., 2012). Group membership can enable health and well-being by providing people with self-esteem, a sense of belonging and social connectedness, and a sense of purpose, control, and efficacy (e.g., Cruwys et al., 2014; Greenaway et al., 2015). In this way, group membership can impact health and well-being positively, acting as a “social cure” (Haslam et al., 2009). Groups, however, also have the potential to negatively affect people’s health and health-related practices (e.g., by promoting unhealthy norms or by failing to provide their members with adequate social support; Dingle et al., 2015), thereby acting as a “social curse” (Kellezi and Reicher, 2012). This is especially the case when group membership becomes stigmatized. For instance, Stevenson and colleagues (2014) showed that negative stereotypes endorsed by healthcare service providers contributed to a perceived division and “stigma consciousness” among service users, undermining the latter’s trust in the services. The study highlighted that, by implication, addressing stigma to foster a shared identity is a fundamental precondition to establishing positive interactions between healthcare users and providers.

According to the seminal work by Radley and Billig (1996), employing a discursive and rhetorically oriented research agenda allows us to explore how descriptions regarding health and illness come to constitute “the world of inequalities, which appears as the ‘real’, ‘natural’ world” (p. 221). By exploring talk about health and illness, researchers who take a discursive and rhetorical approach to psychological research have provided substantial insight into how social actors employ discursive strategies to manage particular interpersonal concerns. These include, for instance, how individuals orient to everyday practices as “healthy” or not (e.g., De Kok and Widdicombe, 2010; Lamerichs et al., 2009; Wilkinson and Kitzinger, 2000), accountability for health problems and ways of addressing them (e.g., Horton-Salway and Locke, 2010; De Kok, 2009; Wiggins, 2009), and how the concepts of health and illness become interrelated with identity concerns (e.g., Bolam et al., 2003; Guise et al., 2007).

A key implication of these studies’ findings for psychological research regarding health inequalities is that control over health is not simply a matter of individual cognition. Discursive approaches that “build upon material and structural analyses to include an acknowledgement of the agency of social actors” (Bolam et al., 2003: 26) provide a richer understanding by bridging the dichotomy between structure and agency in exploring health inequalities. Such analyses further indicate “the importance of challenging wider structural inequalities, as well as the language of individual responsibility that underlies health advice, creating narratives of blame around those who fail to ‘achieve’ it” (Anderson and Gibson, 2018: 333)—an assumption that underlies most mainstream health psychology research, as well as interventions focused on motivating individuals to change their social status and behaviors while ignoring the structural and material setting of their lives (Hodgetts et al., 2020). The present study aims to contribute to discursive research on health inequalities by exploring the ways of accounting for access to health services provided by both professionals and users of health services. By casting light on the accounts of both groups and by adopting a critical discursive approach, the study also aims to link analytic findings with the broader discursive fabric to produce actionable knowledge capable of informing community-oriented approaches that aim to address existing health inequalities. Therefore, it aligns with critical health, community, and social psychology efforts to change some aspects of people’s lived experiences (McVittie, 2006).

## Method

### Background to the study

Greece, the present study’s focus, recorded the second-highest level of unmet healthcare needs in the EU at 8.1% in 2019, compared to the EU average of 1.7% (European Observatory on Health Systems and Policies, 2021). In Greece’s remote regions, thousands of residents are compelled to make long journeys to seek medical care. Several factors impede the ease of healthcare access in these regions, including the sparse health infrastructure, an absence of specialized services in proximity, a low density of general practitioners, and adverse weather conditions that lead to prolonged periods of isolation. Geographical residence constitutes just one among other factors such as income, social class, education, ethnicity, gender, and lifestyle that can explain differences in access to healthcare across diverse social groups (Oliver and Mossialos, 2004). The Mobile Medical Units (MMUs) program, initiated by the non-profit-organization “Regeneration and Progress” in 2014 with the exclusive funding of the Stavros Niarchos Foundation, aims to address precisely this issue by providing citizens of Greece’s remote regions with free-of-charge primary healthcare.

This community-orientated initiative constitutes an influential site for research into the psychosocial processes integral for buffering restrained access to healthcare services. For the study, the first author participated in four MMUs’ missions from April to June 2023 in the following regions: Amorgos and Sifnos (islands of the Cyclades located in the South Aegean region), Lidoriki (a village located in central Greece), and Agios Efstratios (a small island in the northern Aegean Sea). These regions were selected to ensure the most representative sample across regions commonly visited by MMUs within a year.

### Participants and interviews

Ethical approval for the study was granted by the Aristotle University of Thessaloniki Research Ethics Committee (approval no. 76/23–02–2023). A total of 29 semi-structured interviews were conducted by the first author during the MMUs’ missions. The first group of participants consisted of 17 residents from Greece’s remote regions (6 men and 11 women) aged between 29 and 70 years with diverse demographics and socioeconomic backgrounds. The second group of participants consisted of 12 professionals employed by the MMUs (5 women and 7 men), including 3 paramedics, 2 administrative assistants, and 7 physicians. See Table 1 for the sociodemographic characteristics of participants. Informed written consent was obtained from all participants. Face-to-face interviews, lasting between 25 and 75 minutes, were conducted in a quiet space. The interview guide covered residents’ access to healthcare, challenges faced, and their views on MMUs’ services and the Greek National Health System. Interviews were audio-recorded and transcribed for content.

**Table 1.** Sociodemographic characteristics of the participants.

Participants	Gender	Age (years)	Residence
Resident (P1)	Women	42	Amorgos
Resident (P3)	Man	43	Amorgos
Resident (P4)	Woman	53	Amorgos
Resident (P8)	Man	29	Amorgos
Resident (P9)	Man	41	Lidoriki
Resident (P10)	Man	70	Lidoriki
Resident (P11)	Woman	63	Lidoriki
Resident (P12)	Woman	45	Lidoriki
Resident (P19)	Man	33	Agios Efstratios
Resident (P20)	Woman	35	Agios Efstratios
Resident (P22)	Woman	36	Agios Efstratios
Resident (P24)	Woman	61	Agios Efstratios
Resident (P25)	Woman	32	Agios Efstratios
Resident (P26)	Woman	43	Sifnos
Resident (P27)	Woman	47	Sifnos
Resident (P28)	Woman	49	Sifnos
Resident (P29)	Man	52	Sifnos
MMU Professional (P2)	Woman	38	Athens
MMU Professional (P5)	Woman	47	Athens
MMU Professional (P6)	Man	55	Athens
MMU Professional (P7)	Woman	35	Athens
MMU Professional (P13)	Woman	25	Athens
MMU Professional (P14)	Woman	59	Athens
MMU Professional (P15)	Man	59	Athens
MMU Professional (P16)	Man	53	Athens
MMU Professional (P17)	Man	57	Athens
MMU Professional (P18)	Man	50	Athens
MMU Professional (P21)	Man	47	Athens
MMU Professional (P23)	Man	46	Athens

### Analytic procedure

The analysis follows critical discursive social psychology’s theoretical and methodological framework (Wetherell, 1998), which combines a micro-analytic emphasis on the action-oriented nature of discourse with the macro-level focus of post-structuralist approaches on the socio-cultural and historical contexts. Critical discursive social psychology’s key strength when applied within health psychology lies in its ability to document wider societal discourses which influence experiences of health and illness, as well as to underscore how people deploy these discourses within their immediate contexts to achieve particular social aims (Seymour-Smith, 2015). Although this emphasis on situatedness precludes easy generalization across contexts, this does not mean that findings cannot be transferable to other settings and interactions, enabling meaningful insights beyond specific instances (Locke and Budds, 2020).

The first analytic stage involved recurrent readings of transcripts and coding aimed at documenting broader categories of meaning. This procedure was both inductive and deductive, that is, both data-driven and derived from our analytic aims and research questions. It concluded in the identification of two main contradictions/dilemmas that seemed to permeate the accounts of both groups: (a) a contradiction between structural vs individual barriers to healthcare access and b) constructions of belonging to remote small communities as a “cure” versus “curse.” These dilemmas provided the analysis structure and are reflected in the titles of the analytic subsections. In the second stage, the analysis focused on identifying ways of accounting and commonplace representations evoked by interlocutors based on their subject positions. These—abstracted from contextual details—are also used as subtitles preceding the extracts included in the analysis section; at this stage, analysis of specific exchanges attended to the discursive resources used and the subject positions adopted in participants’ effort to navigate the dilemmas mentioned above. Finally, the analysis looked at the micro level of discursive practices (focusing on how representations are constructed to achieve specific interactional goals, such as attributing blame, justifying and accounting). The extracts that follow have been selected for their clarity and density. Although they do not exhaustively cover all topics emerging in the interviews, they are indicative of typical patterns of accounting found in almost all interviews. For the present paper, extracts have been translated from Greek into English.

## Analysis

### Structural versus individual barriers to healthcare access

Both groups’ ways of accounting were complex and multifaceted, prioritizing different explanatory resources. First, residents underscored structural barriers, notably financial constraints, in accessing healthcare services. In justifying reluctance to seek healthcare or follow physicians’ advice, residents drew upon broader consensual representations regarding the status of Greece’s health system, explicitly highlighting the inadequacy of the public health sector and the (financial) inaccessibility of the private sector. Conversely, MMUs professionals, while acknowledging the difficulties geographical remoteness entails, primarily focused on psychological factors, attributing citizens’ reluctance and limited healthcare access to fear, learned helplessness, and personal choice.

#### Accounting in terms of one’s socioeconomic standing

All residents underlined the structural difficulties that may hinder access to healthcare. Here, prompted by the interviewer to elaborate on why she believes certain people are reluctant to seek medical help, a 63-year-old woman narrates her personal experience of having to travel to a nearby city for a medical appointment:

*Extract 1*

Aristi: I mean, it’s all about deterring you… I had a mammogram appointment. I went. The mammograph broke. Why didn’t you let me know? I drove 50 km to get here. I spent money! I live on 200 euros a month! So, nobody’s ever going to think of me? How am I supposed to survive? Where am I supposed to find the courage? And they forced me to get the vaccinations! As if they were feeding me! I don’t want to get the vaccine dude! Why would I get a vaccine? Because you want it? So, you can sell your vaccines? No! I don’t want them! How can you force me? And you’re charging me a fine? (P11, Resident, 63)

Aristi stated during her interview that she is currently divorced, unemployed and in charge of caring for her paraplegic mother. Here, she is narrating a past experience whereby she travelled to another city for her mammogram, only to find out that her appointment was cancelled due to a technical issue. Her question, *“Why didn’t you let me know?”* seems to carry with it the expectation that she should have been informed in time about the cancellation. Explicitly stating her monthly income and the fact that she spent money to travel to get her medical examination hints at her socioeconomic position as a factor impeding her from fully accessing healthcare. In her account, Aristi works to maintain the position of the responsible individual who takes the necessary steps to ensure good health (she booked an appointment and travelled despite her low income). Constructing her negative experience with the healthcare service as an indication of external constraints thwarting her intentions (Edwards and Potter, 1992), her account seems oriented to ward off potential attributions of blame for what could be otherwise constructed as resistance to pursuing a healthy status and enduring whatever difficulty this pursuit may entail.

In the latter half of her account, the participant expresses her unwillingness to get the vaccines for COVID–19, a measure of mandatory validity taken by the Greek state during the pandemic. Aristi refers to vaccination as a measure imposed on her against her will *(“They forced me… ”*). The sentence *“As if they were feeding me!”* serves important rhetorical work, for it informs Aristi’s interlocutors that her compliance is not to be expected when she has received nothing in return. She subsequently constructs the vaccination measure as serving exclusively the interests of others (e.g., *“Because YOU want it?” “So YOU can sell your vaccines?”*), not hers.

Aristi’s account highlights how structural limitations such as one’s socioeconomic standing, the need to travel to another city, and negative experiences of the broader malfunction of the national healthcare services, create the backdrop against which feelings of indignation arise, alongside the belief that contextual factors contradict an individual’s effort to achieve a healthy status. This account exemplifies what has been described as “neoliberal healthism” (Tischner and Malson, 2012: 57); that is, health is spoken as challenging to attain, requiring considerable effort and resources, and thus, as something that can be effectively achieved only from a position of relative privilege. In turn, mistrust toward the national healthcare system emerges as a reasonable justification for citizens’ noncompliance with healthcare measures.

#### Negative representations of public health as impeding compliance with medical advice

The following extract shows how a negative representation of the public hospital is used to justify another resident’s decision not to follow the MMUs professionals’ medical advice. Sofia is a 61-year-old woman who, although born and raised in Athens, has been a permanent resident of a remote island for the last 40 years. Having established the MMUs’ contribution to her region’s community as fundamentally important, here we see Sofia accounting for why she and other residents may “choose” not to follow the medical recommendation received from the MMUs physicians, citing her circumstances as the reason:

*Extract 2*

Sofia: But we don’t go at all, and it is over; we die without going anywhere, we die helpless… For instance, I had the orthopedic surgeons see me, they saw me, they saw me again… they said I must go and get surgery. They said, “You’ll come to Attikon (state hospital in Athens) where it’s free… it’s the best we can do for you… so you don’t have to pay.”

Interviewer: Yeah…

Sofia: …but… I’m not a racist, I swear… but I can’t go to the toilet with seven other Roma women… I wouldn’t want that, because that’s the way it is… […] I’ll stay in this island, helpless and crippled and I’ll lock myself in my house and die inside… that’s what anyone who doesn’t have money gets. Anyone who has money goes to a nice private one, gets his er… knees robotically operated, that is my case, pays his fine money and walks away a gentleman! And you recover quickly with robotic surgery, and you have no problems. Of course, when they saw me, the excellent scientists who came last year told me, “It’s the same thing, don’t be afraid, everything will be fine.” However, I had my doubts because we see other things now that we keep up with the internet; how one person recovers with one thing and how another one recovers with the other… (P24, Resident, 61)

Using first-person plural formulations (“*WE don’t go at all*,” “*WE die…*”), Sofia introduces normativity and consensus to her talk (Edwards and Potter, 1992) and constructs her account as prototypical of other residents’ experiences as well. Turning to a personal example, she informs us that the MMUs’ orthopedic doctors, during their last visit, suggested that she must perform knee surgery and that they recommended the option of a public hospital in Athens, where she could get operated for free. Sofia’s justification for not getting the surgery is of great interest. Following her disclaimer, “*I’m not racist, I swear…*,” she constructs the need to co-exist in a crowded room in the presence of other Roma women as a typical situation prevailing in the public hospital. This representation is, then, used in Sofia’s account to justify her unwillingness to follow the MMUs recommendations (“*I wouldn’t want that…*”), stating further that she would rather stay in her current condition, that is, “*helpless and crippled*.” Although she positions herself as aware of the consequences and responsible for the outcome of her own decision to stay on the island and “*lock*” herself inside, she goes on to construct this decision as inevitably taken by people who, like herself, are positioned in a similar socioeconomic position.

We can see how the constructions of dichotomies (wealthy vs poor, public vs private) in the speaker’s discourse draw upon broader social representations to legitimize her decision, invoking shared representations of the public and private sector and of who is deemed able to access the more patient-friendly procedure, allow the participant to thwart potential accusations of being irresponsible for not taking action to address her medical condition, despite the MMUs’ professionals’ opinion indicating that both procedures are “*the same thing*.” In her account, Sofia positions herself as an informed patient, navigating the available information online and critically evaluating what course of action is best for her health condition. Sofia’s meaning-making of her social standing and economic situation is thus tightly interlinked with broader social representations available to her about the quality of public healthcare services and the (in)accessibility of private ones by the financially disadvantaged.

#### Constructing reluctance to visit healthcare services due to fear and individual choice

Turning now to MMUs professionals, we can see a different picture unfold. Although they seemed to recognize the role of social structures and norms, professionals did not elaborate on how come these factors interfere with citizens’ agency. Instead, their accounts tapped more into individualized reasoning, tailored to explain why part of remote regions’ population does not show up to the MMUs.

Extract 3 involves part of the answer given by an MMUs’ administrative support employee when asked to elaborate on the reasons potentially underlying certain citizens’ reluctance to seek the services provided by the MMUs:

*Extract 3*

Giannis: …there is a category of people who don’t want, they don’t show up because… they don’t want the doctors’ help, they don’t think the doctor will help them. It’s all these people who have basically given up, they’ve come to terms with their problem and […] they’ve given up, they’ve surrendered themselves to a problem, and they don’t do anything […] some people, especially in the areas we visit, they feel that they don’t deserve to be looked after because… that’s what they’ve learned… because that’s what they’re used to, because… that’s how society is…

Interviewer: You mean they feel excluded, and they have accepted that they are excluded?

Giannis: That’s right. And in that category, I put the people that I tell you we don’t even know, who are completely cut off and we’ll never know them, and some people I want to believe are leaving like that, and I say that because through conversations with people in the villages that I’ve done countless times, in talking to children or grandchildren, they say “Oh! I have my grandfather at home who has this problem, but… he will die there…,” this expression ugh… “He will die in bed,” “He will die in the house,” “He will…,” “There is no way he will ever get up to go see a doctor. He will live with his problem.” Ugh… that’s the choice these people make. (P15, MMU Administrative Support)

Giannis provides a range of possible reasons to explain why certain people do not show up to the MMUs visiting the regions where they live. He constructs these people as a category that lacks the willingness to seek medical professionals’ help and the belief that the latter can offer such help. This statement, however, is not further developed; how and why these people came to doubt medical professionals’ help is left unquestioned. The participant portrays this group as having abdicated their responsibility to look after a health problem and mobilize to address it adequately. Giannis’ account subsequently provides a psychological explanation (“*they feel that they don’t deserve to be looked after*”), followed by a three-part list (Jefferson, 1990), consisting of two psychological justifications (“*because… that’s what they’ve learned,” “that’s what they’re used to*”), with only the last part introducing a factor lying potentially outside the individual as a source of influence (“*that’s how society is*”). Accepting as correct the interviewer’s intervention that orients to confirm Giannis’ previous description as compatible with a perceived form of exclusion, he further states that these people are not only “*cut off*” but also impossible for the MMUs’ staff to reach out to and get to know who they are. The use of active voicing of these people’s relatives (Wooffitt, 1992; e.g., “*There is no way he will ever get up to go see a doctor, he will live with his problem*.”) adds facticity to the participant’s account and further supports his argument; people will deny medical help, endure whichever problem they face, and may even die due to it. His final sentence summarizes the above as a “*choice*” these individuals make.

### Belonging in remote small communities as a cure or curse

Most participants’ accounts also provided insightful occasions for understanding how the sense of belonging (or lack thereof) in a remote region’s community may function positively (as a “cure”) or negatively (as a “curse”) in terms of encouraging the pursuit of a healthy lifestyle and enactment of pro-health behaviors.

#### Solidarity and mutual help as a buffer

In Extract 4, a 43-year-old woman, Dafne, despite being invited to discuss whether certain people among the residents of her island face more difficulties in their effort to access healthcare services, she re-directs the focus to the solidarity that exists between remote regions’ residents by highlighting the differences between the latter and big urban centers:

*Extract 4*

Interviewer: …do you identify people here, among the residents of the island er… who are more disadvantaged than others in terms of their ability to access health services? To commute, to go to Athens to have their checkups… er… this, if they need a systematic follow-up…

Dafne: …here in our island, at least I’ll tell you, I guess this applies in other small places too, but I’ll tell you about ours uh… and I think that’s a characteristic of urban centers, to see people completely impoverished, that is, they can’t have access to anything, I think that’s a characteristic of urban centers where you get lost. Here, if a person needs something, society becomes mobilized; that is, it will help you. They may never tell you; they will find money and they will give it to you to go and have your checkup… they will give you food to eat, they will give you a place to stay, they will give you a drink… that is, solidarity exists… (P26, Resident, 43)

Using extreme case formulations, Dafne describes people living in cities as persons who are “*completely impoverished*” and who “*can’t have access to anything*.” Drawing on the increasingly common idea that urban centers are “*where you get lost*,” implying a loss of social relationships, she contrasts this negative representation of society in larger cities to that of “*smaller places*,” referring to the latter as mobilizing in cases where its members need help. Dafne states that in places like her island, the community will go on to offer financial help so that people in need can afford the cost required for a medical checkup, besides getting food and a “*place to stay*.” Likewise, her construction of smaller regions’ societies as socially engaged and keen to offer help draws on a commonsense understanding of smaller communities as places where “*solidarity exists*.” In this account, the island’s community is presented as enabling its residents, no matter their socioeconomic standing, to achieve a healthy status through actions that exemplify solidarity and care.

#### Stigma against illness as a “curse” of living in small-scale communities

While interviewees’ accounts highlighted instances wherein membership to the community of a remote region may constitute a “social cure” (Haslam et al., 2009), fertilizing the ground for pro-health behaviors to occur, in most instances, their accounts revealed the opposite picture. Other characteristics of remote regions’ social landscape, such as stigmatized views against illness and, closely related to this, social exclusion of some members who deal with (mental-)health issues, may negatively influence help-seeking, as well as support-giving behaviors. Thus, when belonging to a remote region’s community ceases to be a source of positive identification, it could be considered a form of social “curse” (Kellezi and Reicher, 2012).

The account provided in Extract 5 exemplifies precisely this latter case, where the articulation of stigmatized views against illness within remote regions’ communities may deter people from seeking medical help:

*Extract 5*

Interviewer: …uh… I just want to ask whether you know from hearsay, from experience… uh… anything, uh, if you’ve heard of people being afraid to get screened or being… uh… distanced a little bit from the…

Maria: Yes… I don’t know if it’s fear that much, but what does exist is the taboo against illness…

Interviewer: Hmm…

Maria: …I mean, I’m trying to understand why sometimes we hide it too. If we have something, we hide it; “This person has this, but don’t say it. It shouldn’t get known that I told you… ” There’s that in small communities. Especially in relation to cancer… and in women… I mean, we see and recognize symptoms… this one, uh… she’s wearing a hat; we say, “What’s going on? Did something happen?” Of course, I don’t know if I was suffering from it… okay, I wouldn’t go out to advertise it, but… I don’t know if one should hide it… (P27, Resident, 47)

Maria starts her account by turning down the interviewer’s articulation of the question that positions residents as being afraid to seek medical help (“*I don’t know if it’s fear that much*”). Instead, her account focuses not on fear per se but on the “*taboo against illness*” as a contributing factor. In using the first-person plural formulation (“*…we hide it too*”), the speaker positions herself as affected by the taboo against illness, although she seems puzzled about why she and others behave in that way. She introduces an example of reported speech highlighting that she and other people discuss another person’s illness, despite not wanting to be seen as involved in spreading this news (“*It shouldn’t get known that I told you*”). Maria’s account seems to draw upon the commonsense view that stigma circulates in smaller communities, specifying that these are especially prominent when it comes to women affected by cancer. She further supports this argument by referring again to her and others as capable of recognizing someone’s symptoms; here, the active voicing (Wooffitt, 1992; “*What’s going on? Did something happen*”) adds facticity to Maria’s account, enabling her (and imagined others) to position herself as genuinely worried about another person’s illness. Toward the end of the extract, we see the participant being caught by the horns of a dilemma; she questions whether hiding one’s illness is correct. Nevertheless, she states that if she were the one dealing with an illness, she “*wouldn’t go out to advertise it*,” implying that there might be a fine line between hiding one’s illness altogether and publicly sharing it with everyone.

#### Fear of social exclusion as deterring health-seeking behaviors

In the final extract, we see an account exemplifying how stigmatized views against illness circulating within a community eventually led to the social exclusion of an affected member. Here, Irene, a paramedic who has taken part in over 100 missions of the MMUs, replies to the interviewer’s enquiry about why some residents of remote regions may not visit the MMUs even though they need the healthcare services offered:

*Extract 6*

Irene: I’ve also heard this in the villages—I’ve heard it in the villages, not on the islands, and from old women, that is, 50 to 60-year-old women: “What the village will say about me?” “I have breast cancer… ” I’ve heard that too. And they didn’t go to the doctors, and they died. Because it had metastasized… You know? There is also this… In Pomakochoria,^1^ I met a young lady […] I kept her medical record, and she told me that in the village when they found out that she had cancer in the uterus, […] she was bullied in the village there, and she left, took her husband and her child, and went to another village nearby.

Interviewer: So, when you say bullying… to what behaviors do you refer?

Irene: They considered, she said, that she was no longer a woman… that… they could no longer… these are Muslims too, they have a certain mentality… (P7, MMU Paramedic)

In her effort to explain why some people do not show up to the MMUs, Irene’s account draws upon the representation of stigma against illness circulating within small-scale communities. Irene introduces the reported speech of women in their 50 and 60 s to emphasize their preoccupation with what their village’s community will think of them (once the news of their illness spread), to which she attributes the fact that some of these women did not seek medical help, leaving their health condition unaddressed until some of them passed away. Referencing a specific example based on her encounter with a woman in Pomakochoria, she informs us that once it became known that this woman had uterine cancer, she was “*bullied*” by the village’s community and that, by implication, she was led to leave the village. Prompted by the interviewer, Irene goes on to elaborate on the way this woman was treated; the community questioned this woman’s gender identity, considering “*she was no longer a woman*.” In her account, Irene implicitly acknowledges this community’s stigma against illness as enabling hostile behavior to emerge, leading to the social exclusion of one of its members. She also seems oriented to attribute part of the reasons that led to this woman’s social exclusion to the Pomak community’s religious identity (“*These are Muslims… they have a certain mentality*”) without, however, elaborating further on her account.

Here, our participant’s talk highlights an instance whereby group-based ‘us and them’ distinctions between the “healthy” and the “ill” resulted in the social exclusion of a woman affected by cancer. This account is, thus, revealing two interrelated phenomena: a community’s stigmatized thinking may fertilize hostile behaviors against certain of its members who may be dealing with an illness. By implication, while stigma against illness prevails within a community, leading to exclusionary behaviors, it seems reasonable to suggest that people will have a justifiable reason to hide their symptoms and deny medical assistance, given the social consequences that dealing openly with an illness might entail for their social identity status.

## Discussion

The present study explored how residents of remote regions in Greece and professionals associated with the MMUs account for the challenges faced by the former in addressing their health needs effectively. Given the ongoing imperative for psychologists to engage in research that explicitly tackles the social, structural, and contextual determinants of health inequities (Rami et al., 2022; Thurston et al., 2023), this study exemplifies the application of critical discursive social psychology to shed light on the psychosocial processes operating at both macro and micro levels, that may compromise the effectiveness and overarching impact of healthcare interventions aimed at addressing the differential access to healthcare observed in Greece’s geographical landscape.

Our analytic interest was prompted by accounts of certain individuals’ hesitancy to seek medical assistance despite the availability of free and accessible healthcare services provided by the MMUs. Both groups’ ways of accounting were complex and multifaceted, yet they tended to prioritize different explanatory resources. Residents primarily cited structural barriers to justify their own and others’ hesitancy to seek medical assistance, while professionals attributed residents’ reluctance to seek help or noncompliance with medical advice to individual psychology. These variations, to some extent, were anticipated and justified by the differing contextual positions of participating residents and professionals, leading to varying accountability concerns. However, their significance remains undiminished. Diverse (co)articulations of interpretative resources may have more distal social implications for the studied communities. Recognizing and reflecting on the power dynamics inherent in the distinct positions of subjects within the social and interactional milieu are fundamental aspects of a praxis-orientated critical public health (Bourdieu and Waquant, 1992).

The analysis also revealed common places between the two groups of participants. Specifically, it manifested how living in hard-to-reach areas was constructed as both a “cure” and a “curse” for residents’ capacity to achieve a healthy status, representing remote communities as either enhancing solidarity and mutual support or promoting stigmatization against illness and social isolation. The fear of stigmatization was represented by both residents and professionals participating in the study as a deterrent to residents’ sharing healthcare problems with other members of their community, as well as to their ability to take the steps required to address ill health effectively. Experience of stigmatization is, according to our participants’ accounts, one (albeit critical) factor amongst the complex agents that serve to restrict access to healthcare services.

Our analysis shows that geographical remoteness interacts in complex ways with other structural and social-psychological factors. Specifically, although geographical residence constitutes the prima facie most important parameter challenging access to healthcare, participants’ accounts revealed how structural and material hardship, representations of and previous encounters with the National Health System, social and psychological parameters combine to create unique and highly contextualized barriers to seeking and receiving healthcare. A vicious cycle is created whereby material hardship combined with negative representations and lack of trust towards healthcare providers infiltrate the way people make sense of their potential to access healthcare.

Our research findings have important implications for healthcare initiatives, in the sense that even when citizens of remote regions are allowed to access healthcare services, either those provided by the MMUs or other service providers, this is not sufficient to ensure that people will use and be able to benefit from the range of services made available to them. Reluctance and fear to seek healthcare can then be viewed as a reasonable response if, for instance, citizens are instructed by physicians to follow a therapeutic plan that entails not only considerable financial costs but also the need to endure negative encounters with a health system that is experienced and thought of as incapable to equally and effectively meet the health requirements of citizens regardless of their socioeconomic standing.

The present work is a first step in identifying and understanding this phenomenon. Further research must document how to reverse these processes in local communities. Although our small sample captures some of the diversity of experiences encountered in hard-to-reach regions, it does not exhaustively document the range and impact of all contextual and psychosocial factors that can explain the differential access to healthcare in particular settings such as the ones under study. Nonetheless, the present study provides an example of how a critical health approach may produce a richer understanding of the psychosocial parameters of health inequalities. It must be reiterated that employing a discursive approach does not reduce the significance of the macro determinants of health and inequity. Quite the opposite; our analysis highlights that attunement to the nuanced and diverse ways of accounting—ensuing from social actors’ positionality in the discursive context—allows us to explore how ideology is (re)produced in people’s interactions. As such, our study may be a highly fruitful avenue for future research into how health inequalities are being (re)produced and maintained.

## Notes

1. Villages in north-eastern Greece, primarily populated by the ethnic minority of Pomaks (for a more detailed account see Figgou, 2012).
